# A Huge Hemorrhagic Epidermoid Cyst of the Perineum with Hypoechoic Semisolid Ultrasonographic Feature Mimicking Scar Endometriosis

**DOI:** 10.3390/medicina57030276

**Published:** 2021-03-17

**Authors:** Gina Nam, Sa Ra Lee, Hye Rim Eum, Sung Hoon Kim, Hee Dong Chae, Gwang Jun Kim

**Affiliations:** 1 Department of Obstetrics and Gynecology, Chung-Ang University Hospital, Chung-Ang University College of Medicine, 102, Heukseok-ro, Dongjak-gu, Seoul 06973, Korea; ginanam@caumc.or.kr (G.N.); gjkim@cau.ac.kr (G.J.K.); 2 Asan Medical Center, Department of Obstetrics and Gynecology, University of Ulsan College of Medicine, 88, Olympic-ro 43-gil, Songpa-gu, Seoul 05505, Korea; Hye8383@naver.com (H.R.E.); kimsung@amc.seoul.kr (S.H.K.); hdchae@amc.seoul.kr (H.D.C.)

**Keywords:** endometriosis, epidermoid cyst, perineum, semisolid, ultrasonography

## Abstract

Epidermoid cysts are small, solitary, and slow-growing lesions that rarely appear in the perineum and mostly arise because of trauma. This study examined a huge perineal epidermoid cyst that slowly grew over eight years in a premenopausal woman. Ultrasonography showed that the hemorrhage in the cyst was a semisolid hypoechoic mass, which mimicked endometrioma, and was tentatively diagnosed as scar endometriosis in the perineum after vaginal delivery. This case study highlights the importance of wide surgical excision and histopathologic diagnosis, even with typical ultrasonography and surgical findings.

## 1. Introduction

An epidermoid cyst is a cutaneous or subcutaneous mass that occurs due to the implantation of epidermal elements in the dermis [1]. It usually arises from the invagination of keratinizing squamous epithelium within the dermis [2]. Epidermoid cysts are typically slow-growing and solitary and can vary in size from a few millimeters to a few centimeters [2]. These cysts are commonly located on the face, scalp, neck, and trunk. Occurrences on the perineum are rare [3,4,5]. Perineal epidermoid cysts are formed spontaneously or traumatically, such as after the circumcision of the clitoris or an episiotomy during normal delivery [1,6,7,8,9]. Ultrasonography and magnetic resonance imaging (MRI) can be used as preoperative diagnostic imaging modalities to differentiate a perineal epidermoid cyst from other perineal tumors (lipomas, scar endometriomas, and skin tags). However, the image’s features are varied, which did not correlate well with the histopathological diagnosis. A perineal epidermoid cyst after vaginal delivery showed typical ultrasonographic and surgical findings as scar endometriosis was described. However, wide excision and the histopathologic exam revealed a hemorrhagic epidermoid cyst. This report describes a case of a huge hemorrhagic epidermoid cyst mimicking scar endometriosis in the perineum through ultrasonographic imaging.

## 2. Case

**Figure 1:**
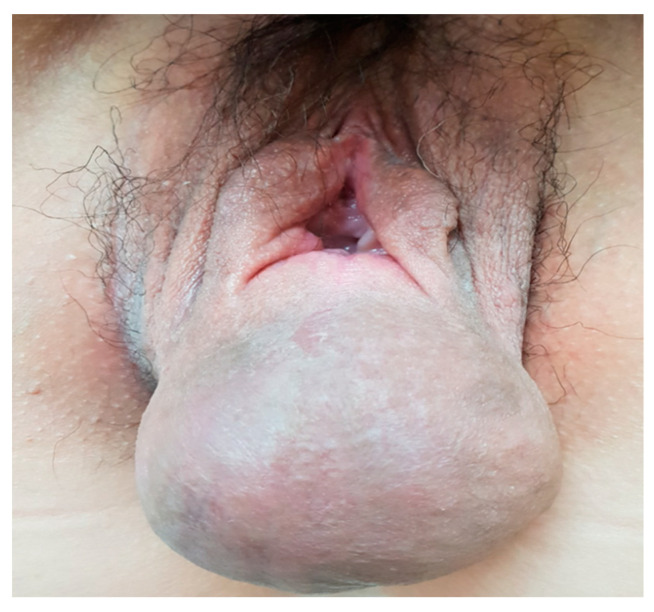
An 8 cm × 8 cm × 6 cm soft, mid-perineal mass without tenderness.

**Figure 2:**
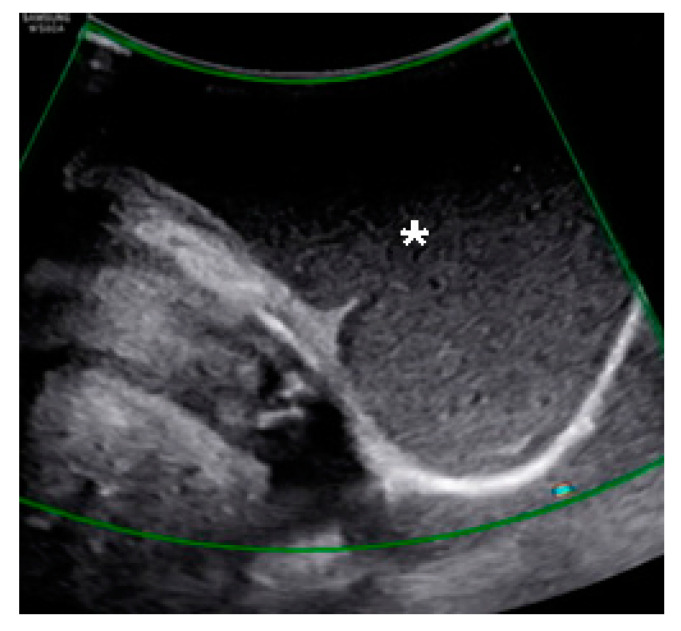
Trans-labial sonography showing a homogeneous hypoechoic semisolid cyst (asterisk) with a “ground-glass” appearance with no flow on the color Doppler imaging.

**Figure 3:**
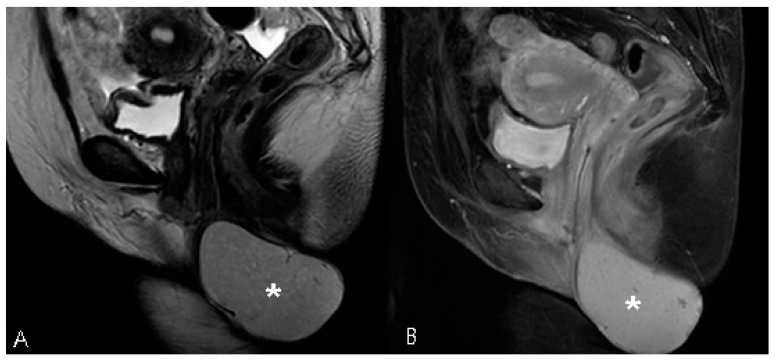
Magnetic resonance image showing a well-defined perineal mass (asterisk) with (**A**) intermediate signal intensity on the T1-weighted image and (**B**) intermediate signal intensity on the T2-weighted image.

**Figure 4:**
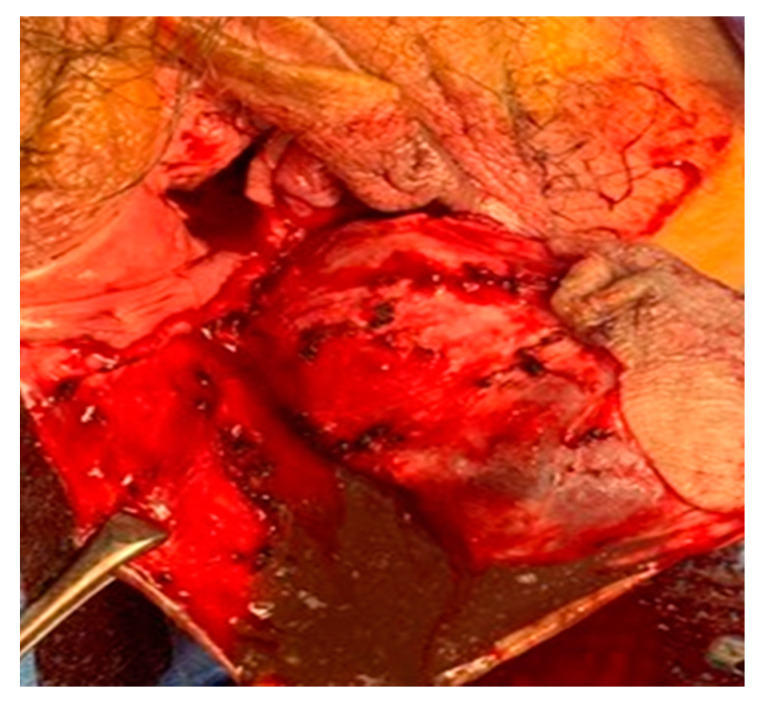
Wide excision showing a thick, chocolate-colored fluid with pale yellow, fat debris.

**Figure 5:**
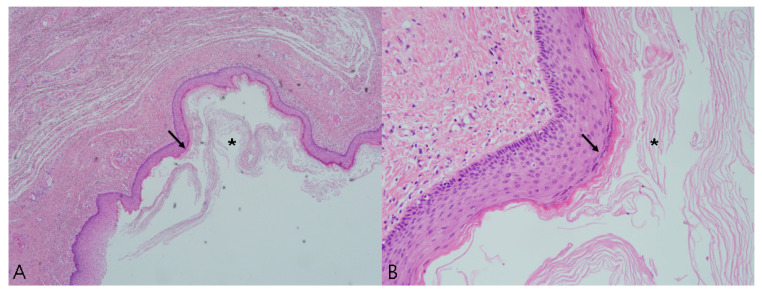
Histopathological findings through hematoxylin-eosin staining showing a cyst wall lined by stratified squamous epithelium (arrow) with a distinct granular cell layer and laminated keratin along the luminal surface (asterisk). (**A**) Original magnification, ×40. (**B**) Original magnification, ×200.

**Figure 6:**
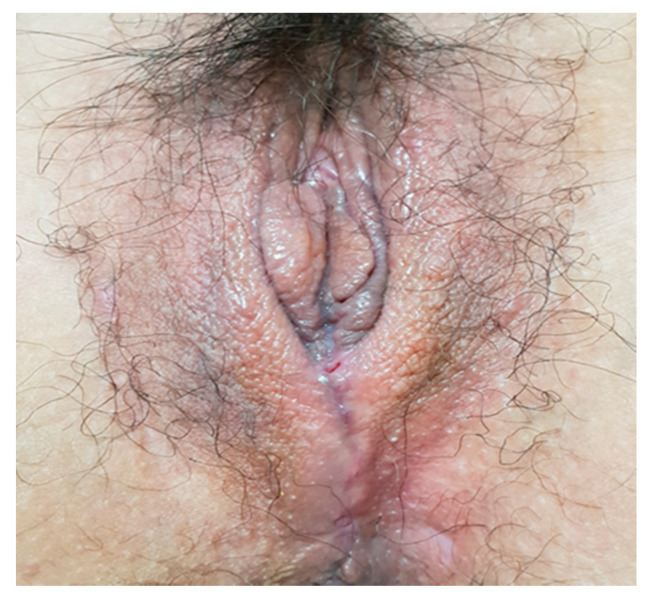
The healed surgical site of the perineum, as observed during the one-month follow-up visit.

A 44-year-old woman was referred for a perineal lump that she experienced for eight years since her last vaginal delivery. No other gynecological symptoms, such as dysmenorrhea and chronic pelvic pain, were reported. Initially, the lump was a painless, peanut-sized mass. It slowly grew to the size of a man’s fist, but its size did not change during the menstrual cycle. The movable gray mid-perineal mass measured 8 cm × 8 cm × 6 cm and was soft without tenderness (Figure 1). Trans-labial sonography revealed a homogeneous, hypoechoic, and semisolid cyst without color Doppler signals. A ground-glass appearance was highly indicative of endometrioma (Figure 2). However, the ultrasound did not suggest any suspicions of ovarian endometrioma or deep infiltrating endometriosis. The high signal intensity on the T1 weighted images (Figure 3A) and intermediate signal intensity on the T2 weighted images on MRI (Figure 3B) confirmed the presence of blood in the mass, which were tentatively diagnosed as scar endometrioma on the episiotomy site. A wide excision and total cystectomy of the mass were performed, and the specimen was sent to the department of pathology for definitive histopathological diagnosis. After a midline incision on the mass, the cyst was carefully dissected. The cystic wall was thick, and a turbid, chocolate-colored fluid, commonly seen in endometriotic cysts, spilled out during dissection. A thick fluid resembling a red-bean paste with tiny, pale yellowish debris, which was less sticky than the chocolate-colored fluid commonly seen in endometrioma, was also noted (Figure 4). After trimming the perineum’s redundant skin with scissors, interrupted sutures with 2-0 Vicryl (Ethicon Inc., Somerville, New Jersey, NJ, USA) were used to close the subcutaneous tissue, layer by layer. A subcuticular running suture was used to approximate the skin layer. Pathology showed an epidermoid cyst lined by stratified squamous epithelium with keratin (Figure 5). On the 1st-month and 6th-month follow-up visits, the surgical site healed, and no recurrence was observed (Figure 6).

## 3. Discussion

An epidermoid cyst is a common, benign intradermal or subcutaneous tumor that slowly arises and grows from the invagination of the keratinizing squamous epithelium [1,2]. It usually occurs on the face, scalp, neck, and trunk, and perineal involvement is rare [3,4,5].

The epidermoid cyst on the female external genitalia is mostly localized on the clitoris following circumcision for cultural or ethnic reasons [7,9]. Clitoromegaly can be the first sign of an epidermoid cyst in the clitoris, and cases with no previous trauma history are scarce [6,7,8]. Two clitoral epidermoid cyst cases have been reported in a 16-year-old woman and a 22-year-old woman with no previous trauma history but who had taken oral contraceptives [6,7]. Paulus et al. [8] also reported a 2-cm clitoral epidermoid cyst in a 47-year-old postmenopausal woman prescribed an estradiol patch without trauma history.

Epidermoid cysts can occur in vulvar lesions, the labia majora, and the labia minora, and large cases without trauma history were usually reported. An 11-cm epidermoid cyst in the labia majora was reported in a 17-year-old girl without trauma history who complained of a painful and palpable mass [10]. In addition, a 15-cm epidermoid cyst in the right labia majora was reported in a 30-year-old woman without a history of pregnancy [11].

Rarely, an epidermoid cyst can occur in the perineum, between the vestibule and anus. The vast majority of cysts are less than 1 cm in diameter and mostly occur because of exposure to trauma, including vaginal delivery. One study reported a case of a 4-cm perineal epidermoid cyst in a 28-year-old woman who complained of a tender vaginal mass on the episiotomy site 10 years after vaginal delivery was reported [12]. In this case study, the patient complained of a perineal mass after vaginal delivery about eight years ago.

Diagnostic imaging tools in the previously reported epidermoid cyst cases consisted of ultrasonography and MRI. The epidermoid cyst’s ultrasonographic appearances vary because the cyst’s contents are diverse, ranging from hypoechoic to hyperechoic lesions. Turkay et al. [3] depicted the ultrasonographic finding as a hypoechoic mass lesion with posterior acoustic enhancement. However, Lee et al. [13] described it as a hypoechoic mass containing variable echogenic foci without color Doppler signals. Meanwhile, another study reported an epidermoid cyst seen as a hyperechoic mass with dense acoustic shadowing [14]. In terms of the perineal cancerous lesion, it is usually hyper-vascular on color Doppler flow imaging [15].

The characteristics of MRI also depend on the composition of the cyst. Thus, variable signal intensity and enhancement of the T1 and T2 values can be seen [16]. These ultrasonography and MRI findings make differentiating an epidermoid cyst from other tumors challenging.

The perineal tumor, along with Bartholin’s cyst, lipomas, endometrioma, post-traumatic hematoma, malignant tumors, liposarcomas, and endometrioma-originating clear cell carcinomas, should be considered during differential diagnosis [15,17,18,19,20,21]. The homogeneous hypoechoic cyst findings showed a ground-glass appearance in trans-labial ultrasonography, and the accompanying hemorrhage in the cyst caused the semisolid sonographic feature to appear similar to an episiotomy site scar endometrioma. The high signal intensity on the T1-weighted images with an intermediate signal intensity on the T2-weighted MRI images is also a confounding factor with an episiotomy site’s scar endometrioma. The chocolate-colored fluid found during excision can also mimic scar endometriosis. Here, the presumptive diagnosis of episiotomy site scar endometrioma was made by a gynecologist with more than 18 years of experience (L.S.R.), including surgical experiences with scar endometriosis occurring at episiotomy sites and cesarean section scars. Most gynecologists can assume this type of mass as scar endometrioma or lipoma. Therefore, pathologic confirmation should be considered. Although the epidermoid cyst’s pathology is rarely encountered, differential diagnosis of the perineal tumor should be accurate for further evaluations and appropriate treatments per case.

For the clinical presentation of the perineal epidermoid cyst, various demonstrations were reported. Some epidermoid cyst cases can be asymptomatic, while, in other cases, the only symptom is a gradual increase in size [7,22,23]. In contrast to the tender scar endometrioma, which can cause recurring pain or swelling according to the menstrual cycle, the epidermoid cysts are usually soft and not tender. The patient may complain of difficulty when walking or sitting, caused by the size’s effect [19]. In this case study, the patient complained of dull perineal pain due to the growing mass’s large size, and the intracystic hemorrhage also contributed to the onset of dull pain. This continuous dull pain was different from cyclic pain or swelling, which are the typical clinical manifestations of scar endometriosis.

Some cases accompany complications, and Nigam et al. [1] reported that 10 out of 103 cases of epidermoid cysts had complications, such as inflammations, ulcerations, ruptures, brown-black melanin pigment collections, and keloid scar formations. When all studies obtained from PubMed were reviewed retrospectively using the terms “epidermoid cyst,” “hemorrhage,” and “perineum,” there were no reports of hemorrhagic epidermoid cysts on the perineum. Moreover, intracystic hemorrhage was rarely reported in other organs, such as the brain and spleen [24,25]. Thus, it was assumed that, when the patient repeatedly sat and walked with an enlarged epidermoid cyst, trauma caused inflammation. This study’s case noted that a hemorrhagic epidermoid cyst should be included in the differential diagnosis of perineal masses with a typical hypoechoic, semisolid mass, indicating a hemorrhage on ultrasonography. Wide excision of cysts is an appropriate treatment method, and a definitive histopathological confirmation prevents complications and recurrence.

## 4. Conclusions

This epidermoid cyst of the perineum is the largest one known in current literature. It is necessary to consider epidermoid cysts when a painless, slow-growing perineal mass, especially in a patient with a history of perineal trauma (vaginal delivery), is encountered. Therefore, a pathological diagnosis should be emphasized even in cases that show the characteristic clinical manifestations of typical hypoechoic, semisolid masses on ultrasonography with thick, chocolate-colored liquid, as seen in this case.

## Data Availability

The data presented in this study are available on request from the corresponding author. The data are not publicly available due to privacy and ethical concerns.
